# Supplementary material for: Peptide Linker Affecting the Activity Retention Rate of VHH in Immunosorbents
Source: Biomolecules. 2020 Nov 27;10(12):1610. doi: 10.3390/biom10121610 (PMC7760621; doi:10.3390/biom10121610)
Supplement: Supplementary file 1 [file biomolecules-10-01610-s001.pdf]

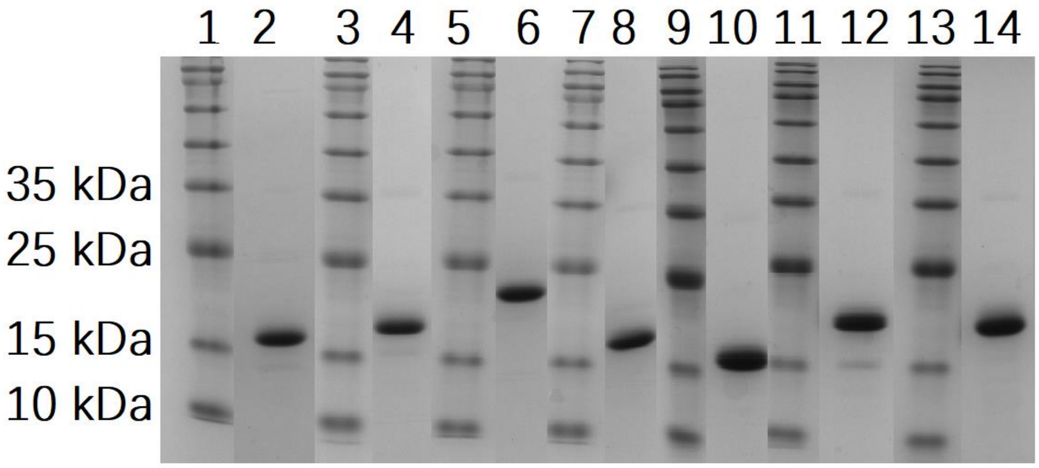

Figure S1 Purification of VHHs with different linkers. Lane1,3,5,7,9,11,13, protein ladder. Lane 2, CNb1-A. Lane 4, CNb1-B. Lane 6, CNb1-C. Lane 8, CNb1-D. Lane 10, CNb1-E. Lane 12, CNb1-F. Lane 14, CNb1-G.

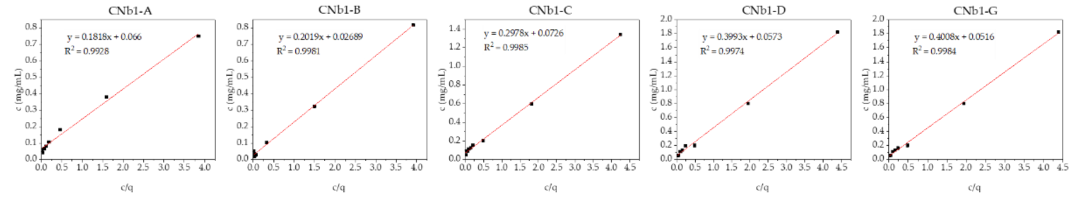

(a)

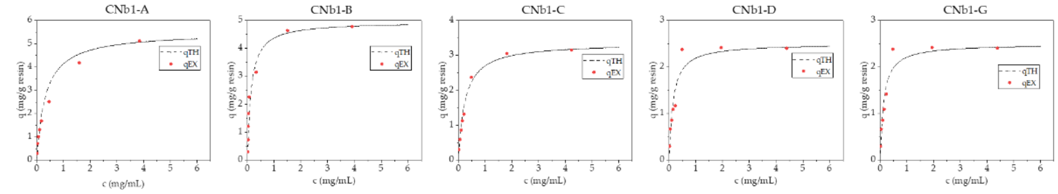

(b)

Figure S2. The static adsorption performance of the VHH immunosorbent. (a) The rearranged linearization curves. The slope of the curve was the maximal adsorptive capacity of the gel, and the intercept of curve was the opposite number of *Kd*. Y represents the equilibrium concentration of β2MG; X represents *c*/*q* (see Equation 2 in method 2.9). (b) The isothermal adsorption curve of the adsorbent at room temperature; qEX represents the test data while the qTH represents the theoretical data.

Table S1. Biacore analysis of the VHHs with different linkers.

VHH	*ka* (10^5/Ms)	*kd* (10^{-2}/s)	K_D (nM)
CNb1-A	7.27±1.36	2.23±0.96	30.93±13.37
CNb1-B	5.03±1.34	2.52±0.36	51.13±5.57
CNb1-C	12.18±6.51	3.51±1.19	31.13±6.77
CNb1-D	12.13±2.25	2.96±0.25	24.73±2.31
CNb1-E	10.90±4.06	1.58±0.90	13.59±3.42
CNb1-F	6.11±0.54	2.30±0.24	37.57±0.91
CNb1-G	4.17±2.19	1.84±0.82	45.43±3.23
